# Successful Treatment of Bladder Outlet Obstruction With GreenLight Laser Photoselective Vaporization of Prostate Operation for a High-Risk Patient With a Very Large Prostate Volume

**DOI:** 10.7759/cureus.72389

**Published:** 2024-10-25

**Authors:** A B Azharul Islam, Maisha Zaman Poushi

**Affiliations:** 1 Urology, West Middlesex University Hospital, London, GBR; 2 Surgery, Dhaka Medical College and Hospital, Dhaka, BGD

**Keywords:** benign prostatic hyperplasia (bph), bladder outlet obstruction, greenlight laser pvp (gll.pvp), high-risk patient, urinary retention (ur)

## Abstract

A 72-year-old high-risk male presented to our hospital with severe bladder outlet obstruction (BOO) caused by an enlarged prostate with a volume exceeding 130 cc. Subsequently, the patient successfully underwent GreenLight laser (Boston Scientific, Marlborough, MA, USA) photoselective vaporization of the prostate (PVP). The patient had been unsuitable for conventional surgical modalities such as transurethral resection of the prostate (TURP) or open prostatectomy due to multiple pre-existing comorbidities, including coronary artery disease (CAD), atrial fibrillation (AF), and concurrent use of antiplatelet therapy. The GreenLight PVP procedure was uneventful and significantly improved urinary symptoms and the patient's quality of life (QoL). This case demonstrates the effectiveness of GreenLight laser PVP as a minimally invasive and effective treatment option for high-risk patients with large prostate volumes (>100 cc).

## Introduction

Benign prostatic hyperplasia (BPH) is a common condition that affects a significant number of men, and its incidence increases with age [1]. The consequences of this condition, recognized as lower urinary tract symptoms (LUTS), have a significant and rapidly deteriorating impact on the daily functioning and quality of life (QoL) of the patient [2]. Failure to address BPH can lead to urinary retention (UR), resulting in bladder dysfunction, reduced flow rates, renal insufficiency, and urinary tract infections (UTIs) [3-5]. Notably, a randomized trial revealed that 2.9% of men with moderate BPH symptoms who chose watchful waiting eventually developed UR [5,6].

The primary treatment for UR in men encompasses using alpha-blockers, 5-alpha-reductase inhibitors, and anticholinergics as monotherapy or combined [7]. In cases where medical management proves ineffective, surgical intervention may be favored, particularly in men with UR, where it accounts for the primary indication of surgery in 24% to 42% of cases of BPH [8]. While transurethral resection of the prostate (TURP) has historically been the gold standard for BPH surgery, minimally invasive laser-based procedures such as holmium laser enucleation of the prostate (HoLEP) and GreenLight (Boston Scientific, Marlborough, MA, USA) photoselective vaporization of the prostate (PVP) have emerged as increasingly preferred alternatives owing to their enhanced safety profiles and comparable functional outcomes [5,9]. This case report describes a successful treatment of bladder outlet obstruction (BOO) in a high-risk patient with a very high prostate volume (>130 cc) using the GreenLight laser PVP.

## Case presentation

A 72-year-old male was referred to the urology clinic at West Middlesex University Hospital in London due to worsening LUTS symptoms, including urinary frequency, urgency, nocturia (four to five times per night), incomplete evacuation, and poor urinary stream despite taking medical therapy with alpha-blockers and 5-alpha-reductase inhibitors for BPH. Later on, the patient developed UR, requiring catheter insertion. The patient failed his trial without a catheter (TWOC) subsequently, so he was on a long-term catheter till his operation. He was deemed a high-risk patient for his significant cardiac history, severe obesity (BMI: 38), hypertension, and type 2 diabetes mellitus. He was on dual antiplatelet therapy (aspirin and clopidogrel) for his CAD. Given his significant comorbidities, he was identified as a high-risk candidate for conventional TURP or open prostatectomy operations.

**Figure 1:**
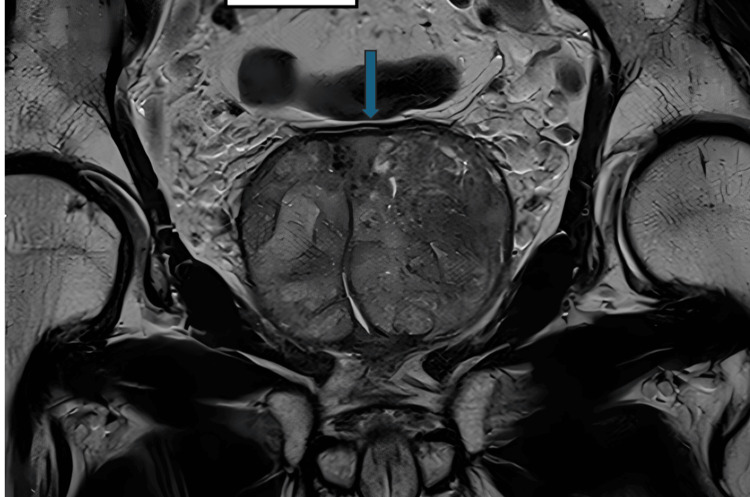
Coronal view of the MRI demonstrating bi-lobar enlarged prostate (blue arrow)

**Figure 2:**
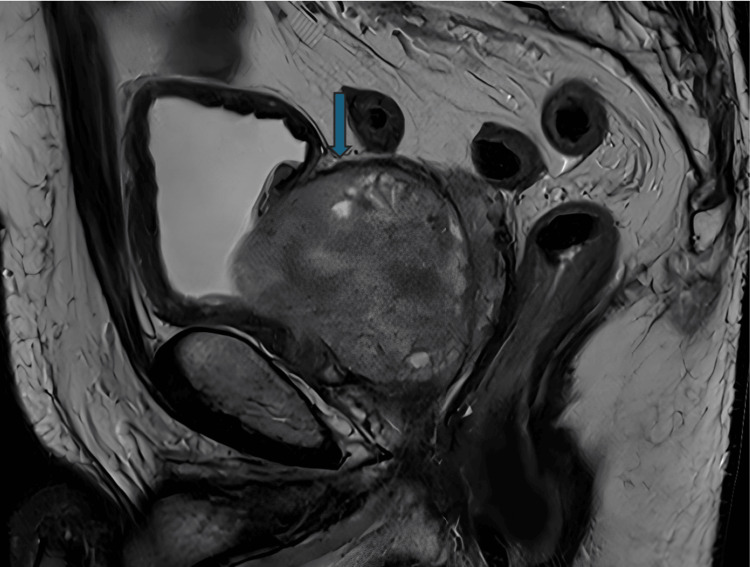
Sagittal view of the MRI demonstrating bi-lobar enlarged prostate (blue arrow)

A digital rectal examination and an MRI prostate (Figures 1-2) showed a significantly enlarged prostate (134 cc). His preoperative international prostate symptom score (IPSS) was 26 (severe symptoms). In addition, his uroflowmetry showed a maximum flow rate (Qmax) of 6 mL/s with a high post-void residual (PVR) volume (550 mL).

The patient underwent PVP using a high-power 180-watt GreenLight laser, which lasted approximately 95 minutes. The intraoperative period was uneventful except for minimal bleeding, and there was no capsular perforation or bladder injury. A 22Fr 3-way catheter was placed at the end of the procedure, and the patient was transferred to the recovery room.

The catheter was removed on the first postoperative day, and the patient passed his TWOC and could void spontaneously with a strong urinary stream. At the eighth-week LUTS clinic follow-up, the patient confirmed significant improvement with his LUTS and QoL, having an IPSS of 7 (mild symptoms). His uroflowmetry showed a maximum flow rate (Qmax) of 18 mL/s with a low PVR volume (30 mL).

## Discussion

The GreenLight PVP treatment has been shown to effectively help with the urinary symptoms associated with BPH. Strong evidence suggests that compared to TURP, GreenLight laser treatment is associated with shorter hospital stays, reduced postoperative catheterization, and higher preservation of ejaculatory function after 12 months. Leading clinical experts have confirmed that, based on their extensive experience, GreenLight laser PVP is a highly effective treatment option for individuals with BPH [10].

In high-risk groups, such as individuals with UR and prostates larger than 100 ml and those at an increased risk of bleeding, the clinical evidence strongly supports the claim that GreenLight PVP is as effective as TURP operation [10]. Laser therapies offer a new approach to treating BPH, and GreenLight PVP is increasingly being scrutinized as a potential primary treatment. This method typically entails utilizing a 532 nm green laser generated with potassium-titanyl-phosphate (KTP) or lithium triborate crystals [11]. The green laser's ready absorption by soft tissue hemoglobin enhances coagulation and diminishes the risk of deeper tissue injuries during vaporization, setting it apart from other laser modalities [12,13]. The GreenLight PVP is a safe and potent treatment modality for treating an enlarged prostate. According to the Goliath trial, GreenLight PVP has been proven to be as effective as TURP in treating this condition [14].

## Conclusions

GreenLight laser prostatectomy is safe and effective for high-risk patients and patients with BOO due to BPH. It has shown low morbidity, short hospital stay, and catheterization time. The operation immediately improves urinary symptoms with a high catheter-free rate. In this case report, the GreenLight PVP procedure is demonstrated as a safe and effective treatment for BOO, especially in a high-risk patient with huge prostate volume and UR.
